# Saturation of the Human Phenome

**DOI:** 10.2174/138920210793175886

**Published:** 2010-11

**Authors:** Mark E. Samuels

**Affiliations:** Centre de Recherche de Ste-Justine, 3175, Côte Ste-Catherine, Montréal QC H3T 1C5, Canada

**Keywords:** Human genome, phenome, genetics, saturation mutagenesis.

## Abstract

The phenome is the complete set of phenotypes resulting from genetic variation in populations of an organism. Saturation of a phenome implies the identification and phenotypic description of mutations in all genes in an organism, potentially constrained to those encoding proteins. The human genome is believed to contain 20-25,000 protein coding genes, but only a small fraction of these have documented mutant phenotypes, thus the human phenome is far from complete. In model organisms, genetic saturation entails the identification of multiple mutant alleles of a gene or locus, allowing a consistent description of mutational phenotypes for that gene. Saturation of several model organisms has been attempted, usually by targeting annotated coding genes with insertional transposons (*Drosophila melanogaster*, *Mus musculus*) or by sequence directed deletion (*Saccharomyces cerevisiae*) or using libraries of antisense oligonucleotide probes injected directly into animals (*Caenorhabditis elegans*, *Danio rerio*). This paper reviews the general state of the human phenome, and discusses theoretical and practical considerations toward a saturation analysis in humans. Throughout, emphasis is placed on high penetrance genetic variation, of the kind typically asociated with monogenic versus complex traits.

## INTRODUCTION

The phenome is the complete set of phenotypes resulting from genetic variation in populations of an organism [1-4]. The concept of phenomic saturation cannot be approached without a careful consideration of the meaning of phenotype, and the genotype-phenotype map. In the most general sense, the phenotype of an individual organism is the sum total of its physiology. Such a broad definition is not particularly useful. In the context of genetics, phenotypes may be defined more specifically as those physiological traits which vary measurably as a function of genomic sequence differences among individuals in a population. Thus, many aspects of physiology can be ignored depending on the type of sequence differences being studied. For example, mutations in the gene encoding the low density lipoprotein receptor (LDLR) have huge impact on plasma cholesterol, atherosclerosis, heart disease, but have no obvious or known impact on eye color or height. In contrast, mutations in the genes encoding pigment-producing enzymes in the iris may be irrelevant to heart disease. One of the advantages of forward genetics is that one begins with an unusual physiological trait observed in actual organisms (human or model animal), and proceeds to search for genetic variation that underlies that trait. In contrast, if one starts with a gene of interest, and mutates it in a directed way (or even through screening undirected libraries of mutants), one has no idea what the physiological effects might be so one is obliged to explore all possible phenotype consequences – a time consuming and difficult task with animals that cannot tell us how they are feeling! Both approaches have been employed in model studies, but human genetics in intact organisms is generally restricted to forward genetics except in some special cases of clinical trials involving somatic cell genetic manipulation (*i.e.* gene therapy).

## MONOGENIC VERSUS COMPLEX PHENOTYPES

**Fig. (1). Monogenic versus complex phenotypes.**
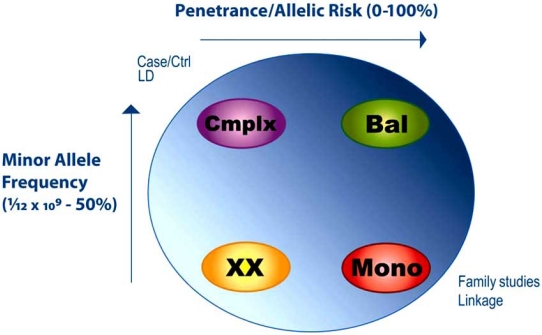
Genetic variants may be anywhere between vanishingly rare (with a minor allele frequency at a minimum of one in the entire human population of approximately 6 billion, thus an allele frequency of 1/12x10^9^ chromosomes) up to 50% (after which the minor allele becomes the major allele). The physiological effect of a genetic variant may be individually very strong (high penetrance) or weak (low penetrance). Rare high penetrance alleles have historically been identified in families, and studied by genome-wide scanning with dense polymorphic anonymous markers, currently SNPs, followed by statistical linkage analysis and gene resequencing to identify causal variants in the family. Common low penetrance alleles are typically identified in large case/control cohorts, and studied by genome-wide SNP genotyping followed by simple statistical tests of differential allele frequency in the two classes, or by more sophisticated linkage disequilibrium (haplotype) mapping. Common high penetrance variants are unusual, since these would normally be either quickly fixed or eliminated from breeding populations. One circumstance under which such variants can be maintained is balanced selection, where there are opposing selections on heterozygotes versus homozygotes (malaria and sickle cell anemia being the best documented example). Rare variants of small functional effect are easily discovered through random resequencing but difficult to study mechanistically.

To some extent, the distinction between monogenic and complex phenotypes is operational. All genetic variants in nuclear chromosomes are *de facto* transmitted in a Mendelian fashion. Thus even weakly penetrant variants, such as most SNPs found by association through large scale genome-wide studies in case control cohorts, are nonetheless “Mendelian”. Monogenic or Mendelian conditions are defined as being caused by variants in single genes, with the variants having a high penetrance (*i.e. *added risk). As a simple example, the clinical phenotype of cystic fibrosis has been made for many thousands of patients across all human populations. In all cases that have been studied, mutations are found in a single protein-coding gene, CFTR. Thus there is a clear one gene-one phenotype correlation for this condition. In contrast, oligogenic or polygenic or complex phenotypes are usually thought of as those for which combined input of many different genetic variants is required, potentially with significant environmental factors required as well (Fig. **1**).

Even for nominally monogenic disorders the case of CF is far from typical. There are many medical examples in which a particular clinical diagnosis has been linked to high penetrance genetic variation in many different genes (e.g. ataxias [5], sensorineural deafness [6-8], retinitis pigmentosa [9-11]). In Charcot-Marie-Tooth disease, causal genes have been found with a broad spectrum of cellular processes including specialized neuronal structures, protein turnover, vesicle fusion, microtubule transport, transcription factors, and even several tRNA-synthetases [12-19]. Clearly, some disease diagnoses represent a common end-point for different types of defects in gene function.

Somewhat less common in human genetics are examples of different allele-specific physiological outcomes. For obvious quantitative traits, such as plasma cholesterol, there are alleles in genes such as PCSK9 and APOB which may alternatively cause hyper- or hypocholesterolemia. These appear to correlate with gain or loss of function, such that loss of function alleles cause either high end or low end mean values in the measured trait, but not both, whereas gain of function alleles cause the opposite mean value. In a developmental example, Huntington disease is caused by triplet repeat expansions of a particular region of the encoding gene leading to some form of aberrant protein which causes neuronal toxicity through an incompletely understood mechanism. Formally such an allele is a clear gain of function (more specifically, a neomorph or new function versus a hypermorph or higher level of normal function). The alternative type of allele, a loss of function, is not known for the HD gene in humans, however the orthologous HD gene directed knockout is lethal in homozygous mice [20], and it is likely that a human loss of function allele would have a similar extreme phenotype clinically unrelated to Huntington disease.

There are also a variety of relatively well-documented examples of digenic inheritance in human disease, such that a clinical phenotype depends on simultaneous genetic variation in two different protein-coding genes. Retinitis pigmentosa resulting from simultaneous heterozygous mutations in the genes peripherin/RDS and ROM1 is perhaps the first documented example [21-28]. There are also likely digenic models for hemochromatosis [29, 30], Bardet-Biedl syndrome [31-36], sensorineural deafness [37-46] and other phenotypes.

For many monogenic genetic disorders there is significant inter-patient variability. This may be due to allele specific effects of the primary causal gene, or to secondary genetic modifiers [47, 48]. In some cases, it can be attributed to well-understood environmental factors. For example, mutations of the gene encoding phenylalanine hydroxylase cause childhood onset phenylketonuria, but there is great variation in clinical severity among patients [49], and disease progression depends on dietary intake levels of phenylalanine, such that dietary restrictions have beneficial effects in clinical practice. Similarly, hereditary hemochromatosis is generally more severe clinically in males than females carrying the same risk alleles; this difference is generally interepreted as females being partially rescued from iron overload through regular blood loss during menstruation [50]. It is widely assumed that combinations of genetic and environmental factors play a major role in clinical severity for many monogenic disorders [51-53]. This has been difficult to address experimentally for most such disorders, as they are individually rare and affected patients are dispersed across the globe. Significant attempts have been made to identify genetic co-factors or modifiers for cystic fibrosis [54-57] and hemochromatosis [58, 59]. The distinction between digenic (or oligogenic) inheritance and genetic modifiers is somewhat semantic, depending on the ease of clinical ascertainment and severity of the physiological effect of the primary gene. In any case, the ability to combine spontaneous, mutagen-induced, and engineered genetic variants in model organisms such as mouse and fruit fly, make it clear that physiological states can be readily decomposed into the effects of multiple genes acting in concert in an individual. The commonly used term “synthetic phenotype” in *Drosophila* genetics refers to the same phenomenon as “digenic inheritance”, or “major genetic modifier”, as used more often in human genetics.

Monogenic disorders are operationally identified primarily by their observed transmission in families. In the case of weakly penetrant genetic variants, or those with major genetic modifiers, it is difficult or impossible to trace the inheritance of the physiological state (whether a disease, a quantititative trait, or a benign trait such as height) in families, in a fashion consistent with a single chromosomal locus. Furthermore, for every anecdotal case of traits “running in families” such as hair or eye color or height, there are examples where this does not occur. Clearly, some physiological states require multiple genetic inputs of similar strength penetrance, and some allelic variants on their own have little physiological impact. Assuming that all physiological traits can be defined by some quantitative variable or variables, although these may not be known, the effect of an individual variant allele can be visualized by its effect on the mean trait measurement. The further from the population mean a particular allelic variant is, the easier it is to detect and follow in individual pedigrees. The distinction between dominant and recessive acting alleles, while a practical complication in the detection of familial transmission, is theoretically not relevant to this point.

Even so, examples are known which blur these distinctions. Age-related macular degeneration (ARMD) has been strongly associated with genetic variation in the gene encoding complement factor H (CFH) [60-63]. The gene was identified independently by family-based linkage and in genome wide association methods, although the GWAS approach was clearly stronger. However, there is a very strong age-of-onset effect, which combined with the natural human life-span makes it difficult to observe familial transmission of the phenotype. Factoring in the age-of-onset, the penetrance of CFH variants is extremely high, and this can be arguably considered a monogenic trait in the affected patients. If humans lived several hundred years, large dominant pedigrees for ARMD caused by CFH variants might well be ascertained.

Not all traits are diseases, obviously, although diseases receive the most attention from geneticists and are often the most readily ascertained through routine clinical surveillance. However, non-medical traits such as hair or eye color, and ability to smell or taste particular chemicals, have been studied genetically [64-70].

## HOW MANY GENES ARE THERE?

Determining the number of actual genes encoded by human (or other) genomes has proved surprisingly vexing. From early estimates in excess of 80,000 protein coding genes in the human genome based on quantitative measurements of CpG islands [71], estimates have dropped repeatedly, to 28-34,000 based on exon comparison between the pufferfish *Tetraodon nigroviridis* and human genomes [72], to 21,037 based on large scale cDNA library sequencing [73, 74], to 20,488 based on comparison of proposed human genes to sequences of other primate genomes [75]. Currently RefSeq, a widely used NCBI library of gene annotations, includes 21,515 unique entries. These include substantial numbers of non-protein coding genes such as 363 small nucleolar RNA (SNO) genes, 28 SNAR genes and 637 micro-RNA (MIR) genes, so RefSeq probably includes approximately 20,000 protein coding genes. There are several ongoing manual gene curation efforts, specifically Havana, VEGA [76, 77] and CCDS [78], as well as the Mammalian Gene Collection (MGC). The CCDS has currently curated 18,173 different protein coding genes, although some well documented genes are visibly missing from the database which is thus still in progress. The MGC currently includes 17,592 genes for which full length cDNA clones are available.

Although a consensus appears near that the total count of protein coding genes in the human genome is between 20-22,000, the advent of next-generation technologies has allowed further exploration of alternative splicing through deep resequencing of cDNA libraries. Several recent studies have documented novel alternatively spliced forms of known genes, as well as completely novel exons within previously studied genes [79, 80]. Thus the number of potential alternative protein isoforms is many times greater than the actual gene count.

The human mitochondrial genome has been comparatively straightforward to annotate, as it resembles bacterial genomes in gene structure. It encodes 13 proteins, all involved in respiratory electron transport and oxidative phosphorylation, as well as 22 mitochondrion specific tRNAs, and the 12S and 16S ribosomal RNAs (rRNAs) required for the mitochondrial ribosome [81]. However, the human mitochondrion itself contains 900-1000 different proteins, based on direct proteomic analyses [82, 83]. Thus the large majority of mitochondrial proteins are encoded by nuclear chromosomal genes.

## STUDIES IN MODEL ORGANISMS

Saturation mutagenesis of model organisms has been widely used to identify mutable genes either for viability or for various interesting biological phenotypes [84]. In diploid organisms, dominant mutations are readily detected in heterozygotes after mutagenesis and breeding to rule out direct effects of the mutagen, although dominant lethality is obviously a difficult phenotype to recover. Recessive mutations can be detected in fungi by sporulation and reduction to haploidy, or in metazoans by establishing mutagenized heterozygous lines and inbreeding to create homozygotes, or particularly in *Drosophila melanogaster *by complementation testing of mutagenized heterozygotes with chromosomal deletions of cytologically or molecularly defined extent. A longstanding question has been whether all biochemically defined genes (transcribed, spliced, protein coding/noncoding, etc) have detectable mutational phenotypes. Even now, only a few historically saturated regions can be well-defined in terms of chromosomal extent, number of genetically mutable loci, and annotated protein-coding transcripts. With the advent of the complete genomic sequence plus extensive gene annotation, such studies can be revisited. This brief overview will focus on animals, leaving plant genetics aside.

The budding yeast *S. cerevisiae* is relatively straightforward to saturate since most genes lack introns. As a result at least for protein-coding genes, predictions can be made based on the occurrence of long contiguous open reading frames. Although the lower limit cutoff is somewhat arbitrary, based on annotation of the complete genome sequence the yeast genome project in 2001 identified 6138 potential protein coding genes. Essentially all of these have been individually deleted, and the resulting strains analyzed under a few different sets of growth conditions [85-87]. According to the yeast deletion project, 18.2% of genes are essential for growth in rich medium. A large-scale effort is also under way to map genetic interactions by combining individually deleted genes in pairwise combination [88]. Ultimately over 36 million different pairwise tests must be performed; to date 5.4 million tests have been reported but the results are intriguing. Approximately 170,000 interactions were detected, meaning that a strain carrying both deletions differed significantly in fitness from either single deletion strain. These interactions could be further studied by overlaying orthogonal annotations such as functional cellular modules, expression profiles, and protein-protein interaction profiles.

In *D. melanogaster*, historical saturation mutageneses have been performed for multiple genomic regions, while the essentially complete genome sequence of the organism together with a preliminary gene annotation was published in 2000 [89]. Overall the fly genome is believed to contain 13-14,000 protein coding genes. Saturation of the *zeste-white* region of the X chromosome in *D. melanogaster *has been performed repeatedly, using various mutagens [90, 91]. Defining the region as that uncovered by a particular deletion (Df(1)w^rJ1^, encompassing cytological bands 3A2-3C2), mutations were initially identified in 17 loci, 15 generating lethality and 2 generating visible phenotypes. Currently FlyBase annotates 31 genetic loci uncovered by this deletion, including genes with additional lethal, female sterile or visible phenotypes beyond the original 17. One caveat is that in rare cases, different mutations within the same gene may show genetic complementation, overestimating the number of actual genes. Through transcriptional and comparative genomic analyses, Flybase annotates a total of 43 genes in this chromosomal region, including 19 identified only as candidate genes. In some cases genetically defined loci have not yet been assigned to structurally defined genes. Thus up to three fourths (31/43) of annotated genes may be mutable to an observable phenotype in this region.

In another chromosomal region also intensively studied in *D. melanogaster*, a strikingly different result was obtained. In a 2.9 million base region around the alcohol dehydrogenase (*Adh*) gene, Ashburner *et al.* documented 218 potential protein coding genes, but only 73 genetically definable loci in the region, of which only 49 have been assigned to specific structural genes so far [92]. Thus in the *Adh* region only a third of structurally defined genes may have individual mutational phenotypes.

Thus a substantial fraction, and in some cases a majority of protein coding genes in an interval do in fact generate a mutable phenotype, typically recessive lethality. Attempts at saturation mutagenesis using P-elements have been widely performed in *D. melanogaster *[93], however it is well documented that this transposon shows significant site specificity thus is not a good mutagen for true saturation. Efforts are currently under way to generate genome-wide saturation with transposable elements demonstrating less site specificity than the original P-element [94-96]. Although the larger number of genes in the fly makes whole genome pairwise genetic mapping a daunting prospect, nonetheless *de novo* mutagenesis in genetically compromised fly strains is a routinely used tool to identify genetic interactions and members of shared physiological or regulatory pathways [97, 98]. Such modifier genes are often referred to as suppressors or enhancers of a phenotype, which may be either visible or lethal in nature.

Although the small nematode worm *Caenorhabditis elegans* has a shorter experimental history than flies, ease of manipulation quickly made it a favorite among developmental geneticists. Moreover its small number of cells has permitted the unique opportunity to map all of embryonic and postembryonic development at the cellular level [99, 100]. The *C. elegans* genome has also been sequenced, and annotated to contain slightly fewer than 20,000 protein coding genes [101]. It remains puzzling why the worm, a much ‘simpler’ organism than the fly, has more genes, and in fact has about the same number as mammals. *C. elegans* has been the subject of a large number of genetic analyses, and pilot transposon-mediated mutagenesis has been performed [102]. However, the use of antisense RNA (RNAi), originally discovered in *C. elegans *[103], has permitted large scale gene knockdown experiments using libraries of gene-specific antisense targeting reagents, formally similar to germ-line loss-of-function mutagenesis [104, 105]. A study of RNAi targeting 16,757 genes from a predicted set of 19,427, detected mutant phenotypes for 1,722 probes, or 10% of targeted genes. Of these, slightly more than half caused embryonic lethality (RNAi was fed to hermaphrodite worms, allowing analysis of the effects in their progeny), with an estimated success rate of 70% compared to a set of genes with known germ-line embryonic lethal mutations. This suggests that 8% of genes can mutate to an embryonic lethal state in this organism. The overall low rate of observed phenotypes is difficult to interpret; it may simply be that most genes are functionally redundant in the worm. Alternatively, RNAi reagents may not be effective in completely reducing gene function for some genes.

Large scale random mutageneses have been performed in *Mus musculus* using both chemical and genetic mutagens [106-125]. Chemical mutageneses typically employ ethylnitrosourea (ENU), but the prohibitive logistics of generating and maintaining the huge numbers of heterozygous F1 lines, required to create homozygous F2 animals to screen systematically for recessive mutations, have precluded a true genome-wide saturation. Genetic approaches primarily involve transposon-mediated insertional mutagenesis in mouse embryonic stem (ES) cells. There are now multiple libraries of embryonic stem cells carrying random transposon insertions, for which insertion sites have been mapped by direct sequencing. This potentially permits researchers to study the insertional phenotype for a gene of interest, if it happens to exist in a library. The vast majority of these transposon insertions have not been developed from ES cells into whole animals. Moreover, many of these insertions are not explicit gene knockouts as insertions are not targeted to coding exons.

As an alternative to random mutagenesis, many targeted gene knockout mouse strains have been constructed. These are inevitably biased in favor of “interesting” genes, with the occurrence of known human genetic phenotypes being one component of interest. However there are other reasons for genes to be defined as interesting, and it is unclear whether existing knockouts are biased toward genes certain to yield a detectable mutant phenotype in mice. In a review of mouse knockouts as of 1995, it was reported that of 263 different genes targeted, 25% yielded an embryonic lethal phenotype, with another 10% leading to death within a few weeks postnatally [126-128]. Overall, 95% of knockouts were reported to show an observable phenotype, a very high proportion in comparison to the RNAi results with *C. elegans*. Subsequently it was reported that about 1/3 of direct knockouts yield embryonic lethality [129]. In an analysis of mouse knockouts for 34 genes which are known targets of commercial therapeutics for important human diseases, all but one knockout strain showed some detectable phenotype in the mouse, most of which were directly relevant to the equivalent human disease [130]. In one case, that of insulin, the gene is known to be duplicated in the mouse genome, such that the double knockout is lethal whereas either gene alone does not yield a phenotype [131, 132]. In contrast, a knockout disrupting the insulin receptor gene, which exists in a single haploid copy in the mouse genome, is severely ill and dies shortly after birth, validating that the insulin pathway is intrinsically mutable in the mouse [133, 134].

The zebrafish *Danio rerio* has been the subject of many large scale mutageneses, since it was originally developed as a research organism by Streisinger [135-140]. In the absence of a gene-specific targeting system, most mutageneses have involved ENU followed by screens for interesting biological phenotypes. Identifying the actual mutated genes requires large scale recombinational mapping using anonymous polymorphic DNA markers, either microsatellites or SNPs, followed by positional cloning. Since the advent of high throughput sequencing, an intermediate approach called TILLING has been developed [141-143]. Instead of mapping and positional cloning, mutations in a given gene are found directly by sequencing pools of very large numbers of mutagenized animals (or plants, where the method was originally developed [142]) until a variant in the desired locus is detected. Using these approaches mutations have been found affecting many different physiological systems. Another effective approach in zebrafish is the use of antisense reagents. Modified oligonucleotides containing morpholino groups to reduce enzymatic breakdown can be injected in early fish embryos [144]. Oligos targeting genes are capable of producing very specific knockdowns, similar to the situation in worms. One limitation is that the morpholinos are eventually degraded, so only events of embryogenesis can be interrogated with this technology. Morpholinos have been used in other important experimental organisms such as the clawed frog *Xenopus laevis*, but genomic saturation screens have not been reported.

In sum, mutational studies in model organisms have demonstrated that a sizeable proportion of genes can be mutated to generate observable phenotypes. The exact number is difficult to determine from the available data sets, as there seem to be broad discrepancies between yeast and worm (small proportion of directed knockouts/knockdowns generating phenotypes) and flies (substantial portion of genes mutable) and mice (large proportion of directed knockouts generating phenotypes), nor is there a consensus as to the proportion of all genes that are essential for viability. It remains to be seen how these results will translate to human physiology, although it seems safe to assume that there must be substantial numbers of genes mutating to embryonic lethality, to viability with clinically observable phenotypes, or to complete viability.

## HOW MANY MONOGENIC DISORDERS ARE KNOWN?

Keeping these points in mind, how many characterized monogenic phenotypes are there in humans? The entire human genetics literature is vast. There are special locus-specific databases and/or web sites for many genetic disorders, such as cystic fibrosis (http://www.genet.sickkids. on.ca/cftr/app) and familial hypercholesterolemia (http:// www.umd.necker.fr/LDLR/Home_Page.html). There are at least three organizations attempting to curate the entire literature [145]. The best-known, Online Inheritance in Man (OMIM), functions as a mixed medical and genetic database, with entries for clinical disorders intermingled with entries for specific genes [146]. It has limited direct search functions. Querying with the universal term 0001 together with a limit for allelic variants, OMIM returns 2420 independent gene entries at the time of writing, while the statistics page reports 2763 phenotypes with a known molecular basis; the excess presumably resulting from some genes mutating to different phenotypes with independent OMIM entries [147]. Almost all of these represent monogenic disorders (as practically defined, high penetrance variants with reasonable evidence of genic pathogenicity based on the mutation itself). However, a fraction of these, possibly as many as 10%, may represent low penetrance risk alleles not clearly definable as part of monogenic disorders. OMIM does not curate all reported genetic variants in all genes, although entries are regularly updated when scientifically interesting results are published.

The Human Gene Mutation Database (HGMD) curated at Cardiff University, attempts to consolidate all reported genetic variants in the literature [148]. It has an incomplete publicly available version, and an up-to-date commercial version. The commercial HGMD currently includes 3611 independent gene entries and 96,631 mutation entries. It is difficult to assess how many of these correspond to monogenic conditions, versus low penetrance risk factors for complex phenotypes. HGMD suggests that 2491 genes contain at least one presumptive high penetrance allele (frameshift or premature stop codon, splice site, missense or indel) (*P. Stenson, pers comm*) [149]. The difference of fewer than 100 genes between OMIM and HGMD may relate to subtleties of literature curation of suggested versus confirmed genotype/phenotype causation.

The Human Genome Variation Society (HGVS) is developing a program of comprehensive curation which may eventually provide similar information as OMIM and HGMD combined [150, 151]. Currently the database HGVbaseG2P appears focused primarily on the results of GWAS studies for complex disorders. It includes links to HGMD, but presumably only the publicly available content not the full proprietary database.

The total number of molecularly characterized, true monogenic disorders remains slightly uncertain, but is slightly less than 2500, representing 10-12% of protein-coding genes. For the remaining ~90% of genes, no high penetrance genetic variation has been causally linked to a physiological phenotype in humans.

## MUTATION RATES

Rates of spontaneous mutation in the human genome have been estimated in various ways. Combining early results of sequencing a small number of real or pseudogenes, Drake *et al*. suggested a rate of approximately 10^-8^ events per base pair per generation, equivalent to 64 new mutations in each zygote [152]. By comparing partial human and chimpanzee sequences, Nachman and Crowell estimated a neutral rate of 1.3-3.4x10^-8 ^events per bp per generation, equivalent to 91-238 new mutations per zygote [153]. More recently a direct test was performed by sequencing large segments of flow-sorted Y chromosomes from two males of a precisely known ancestral genealogy. Four new mutations were detected between the two samples, which correcting for chromosome size yielded a rate of 3x10^-8 ^events per bp per generation, although the small number of individual events implies a reasonable sampling bias [154]. Finally, whole genomes were resequenced for a pedigree of two parents and two offspring, allowing the most direct possible measure. A total of 28 confirmed new mutations were detected, which after various corrections yielded a rate of 1.1x10^-8^ events per bp per generation, equivalent to 70 new events per zygote, with a substantially lower chance of sample bias [155].

These all represent essentially point mutation rates. It is now clear that larger structural rearrangements, generally termed copy number variants (CNVs), also arise at reasonably high frequencies in human populations, although it is difficult to assess true *de novo* mutation rates given the somewhat arbitrary definition of a CNV [156-158]. Many CNVs include genes and have potential functional consequences, although the proportion is not yet well established [159].

Not all new mutations need have functional consequences. It is generally considered that most of them are in fact functionally neutral. There is no easy way to assess this since most new mutations arise in non-coding regions of the genome, in either introns or intergenic regions, where our understanding of functional elements is still very incomplete. The fraction of haploid sequence coding for protein is typically considered to be 1-2%, thus if each zygote receives 70 new mutations, at most one of these is expected to be in a coding exon. Coding variants may be silent (synonymous) or change an amino acid (nonsynonymous). Some studies have argued that a large proportion of nonsynonymous changes have functional significance, although these may only be evident over evolution time frames and not be directly relevant to interpretation in individuals [160-163]. It should be noted that synonymous changes may also have measurable effects on gene function, by influencing splicing *via *exon splice enhancer or repressor elements, or translation efficiency.

The rate of new deleterious mutations (usually termed U when summed over the entire genome), while certain to be substantially less than the rate of overall nucleotide change, cannot readily be inferred from the global rate. A comparison of human and chimpanzee sequences, using some assumptions about evolutionary selection, led to an estimate for U of 1.6 per zygote [164]. A separate analysis, again using human/chimp comparison, obtained a value of U = 3 [153]. In a key study, Kondrashov combined and re-analyzed data for 20 different genes for which typical loss-of-function mutations lead to either autosomal dominant or X-linked high penetrance clinically ascertainable phenotypes [165]. Medical ascertainment rates per live birth include both new cases and familial cases, but by restricting to new cases alone, the median and mean total mutation rates for this set of genes can be calculated as 7 and 14 x 10^-6^ per live birth respectively. Assuming 20,000 protein coding genes, these are equivalent to U = 0.14-0.28 per genome per birth, substantially lower than the rates estimated by evolutionary comparison. A likely explanation for the discrepancy is again that evolutionary comparisons probably involve functional effects too modest to yield a clinically ascertainable phenotype in individuals. Although a rate of U ~ 0.2 seems low, given a worldwide 2009 birth rate of approximately 139,000,000 [166], this represents almost 28 million new pathogenic mutations, or 1400 new mutations per gene worldwide. Even Canada, with a modest total population of about 34 million, has an annual birth rate of 378,000, and could have as many as 75,000 new pathogenic mutations per year nationwide. A significant caveat is that these rates may reflect a bias to genes with unusually high mutation rates. A number of X-linked recessive mutating genes have lower mutation rates, such that a value of U = 0.02 may be a much more conservative genome-average estimate [167]. Even so, the entire human gene repertoire is clearly saturated annually worldwide with new pathogenic alleles. There is no shortage of high penetrance mutations in every gene segregating in human populations. The only reason why high penetrance monogenic disorders are relatively rare is that most mutations act recessively.

## DOMINANCE VERSUS RECESSIVITY, GAIN VERSUS LOSS OF FUNCTION

The proportion of monogenic disorders transmitted as dominant versus recessive traits in families is hard to determine from the curated databases. Similarly, it is difficult to assess the proportion of gain-of-function (gof) versus loss-of-function (lof) alleles, although this is not purely an issue of curation since for many alleles the functional effect remains untested biochemically. Complete gene deletions represent the gold standard for a loss-of-function allele, however few clean deletions of individual genes have been associated with monogenic conditions (whereas many contiguous deletion syndromes encompassing multiple genes are well documented). Geneticists generally assume that major protein truncations arising through frameshift or premature stop codon mutations are pathogenic, but it would be naive to presume that these all represent complete loss-of-function since amino terminal protein fragments could easily encode partial or aberrant activity. Wilkie provides a useful compendium of molecular mechanisms that can lead to genetic dominance (haploinsufficiency, excessive gene expression, novel toxic protein function, etc) with examples from model organisms and the human genetics literature [168]. For individual genes, the best way to distinguish between gain and loss of function phenotypes is to observe the array of alleles associated with a particular phenotype. For example, mutations of BRCA1 which increase the risk of early-onset breast cancer include a wide variety of missense, truncating and deletion mutations [169]. It is reasonable to presume that most of these represent loss of function alleles. Similarly, many independent alleles causing Huntington disease all involve expansion of the triplet repeat, while causal mutations elsewhere in the gene are not known for this phenotype, supportive of the interpretation that these are gain of function alleles. In contrast, Friedreich ataxia is typically caused by a triplet repeat expansion in the frataxin gene, however a small fraction of patients result from point alleles, both missense and truncating [170]. Conceivably, the ataxia in general could represent a loss of function phenotype, and the frequency of triplet repeat expansion alleles could result simply from an easily accessed mutational mechanism versus a specific novel function of the mutated protein. Alternatively, some specific point alleles could cause an aberrant novel protein function akin to that of the triplet repeat expansion. One glaring example is the gene encoding superoxide dismutase (SOD), mutations in which are causal for a fraction of cases of amyotrophic lateral sclerosis (ALS, or Lou Gehrig disease). There are many different causal mutations in SOD, almost all of which are missense variants [171]. However, a mouse gene knockout does not generate the ALS phenotype, whereas overexpressing equivalent missense variants recapitulates some aspects of the human disease [172]. The consensus in the field is that many or most of these missense variants, which arise in almost every residue of this small tightly folded protein, are somehow gain-of-function, although the exact biochemical mechanisms are still being elucidated. Thus it cannot be assumed that phenotypes arising from many different mutations in a gene invariably represent loss of function, although they probably do in most cases.

The clinical presentation of different mutant alleles can sometimes clarify the interpretation. Hypercholesterolemia can be caused by one of a small number of missense mutations in the gene encoding protease PCSK9. However, a much larger number of mutations including obligate protein truncations in the same gene are associated with hypocholesterolemia. The hyper alleles are typically heterozygous, whereas the hypo alleles are typically homozygous in the patients [173-177]. These observations strongly suggest that the hyper alleles are gain of function, and the hypo alleles are loss of function. The rarity of the gof alleles is consistent that the map of genotype to the hyper phenotype is more highly constrained in this gene than the map from genotype to the hypo phenotype. Similarly, many presumptive lof alleles of the gene encoding apolipoprotein B exist, and lead to hypobetalipoproteinemia (with low LDL-cholesterol) due to lack of this major structural protein component of VLDL and LDL particles. In contrast, only a small number of specific amino acid missense variants are known in the ApoB gene causing hyperbetalipoproteinemia (with high LDL-cholesterol), with the mechanism believed to be failure of recognition of mutant ApoB protein by the LDL receptor, and thus inability of the body to remove LDL particles from the plasma [178-180].

Although most mutations are probably recessive, the ratio of dominant to recessive monogenic disorders is probably significantly skewed in human genetics in favor of dominants. High penetrance mutations segregating as heterozygotes have the potential to generate large multigenerational pedigrees transmitting the physiological trait. If the trait is a medical disorder, such as Huntington disease or Alagille Syndrome, such large families are relatively obvious to astute clinicians, particularly since the advent of effective molecular mapping in the 1980s. In contrast, recessive disorders often involve only a single affected in a nuclear family, since only ¼ of offspring on average segregate the trait (for an autosomal disorder). Ascertainment of recessive traits by clinicians typically involves unusually large sibships with recurrence of the phenotype in multiple children. In small sibships with only one or a few affected children, environmental or stochastic etiologies are more likely, and the hypothesis of recessive segregation is less easy to demonstrate except by a genome-wide mapping experiment.

There is a strong practical reason to believe that most loss of function alleles function as recessive genetic traits. Measured rates of mutation are consistent with very high rates of occurrence of novel pathogenic alleles in humans, perhaps as many as one per meiosis. If many new mutations are pathogenic, then severe effects on fitness of entire populations would ensue if most mutations were dominant with pathogenic effects in heterozygotes. Hence, most mutations are reasonably anticipated to have recessive effects on physiological traits (even if they have formally co-dominant effects on their immediate biochemical function through simple gene dosage effects). Indeed, protection from high mutation rates is likely one of the evolutionary forces driving diploidy and hence sex, although most evolutionary biologists tend to focus on sex as a mechanism for generating rather than reducing diversity.

**Fig. (2). Saturation kinetics and genetic dominance.**
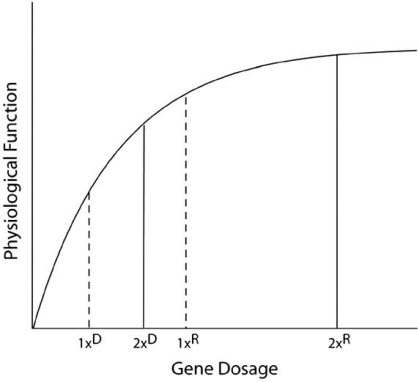
As suggested by Wright, if two doses of a wild type gene in a diploid organism provide saturating levels of physiological function (2x^R^), then loss of one copy through mutation would still provide most functionality (1x^R^). Such a situation would lead to recessive genetic behavior, whereby a heterozygote lof mutation would have little biological impact. Wright hypothesized that this would be the case for the majority of genes. In some cases, two wild type gene doses might provide function in the subsaturing range (2x^D^), such that loss of one dose in heterozygous lof mutation carriers would have substantial biological effect (1x^D^), leading to observed dominance. Dominance caused by unusual gain-of-function alleles is not an element of this model.

**Fig. (3). Metabolic control theory and gene networks.**
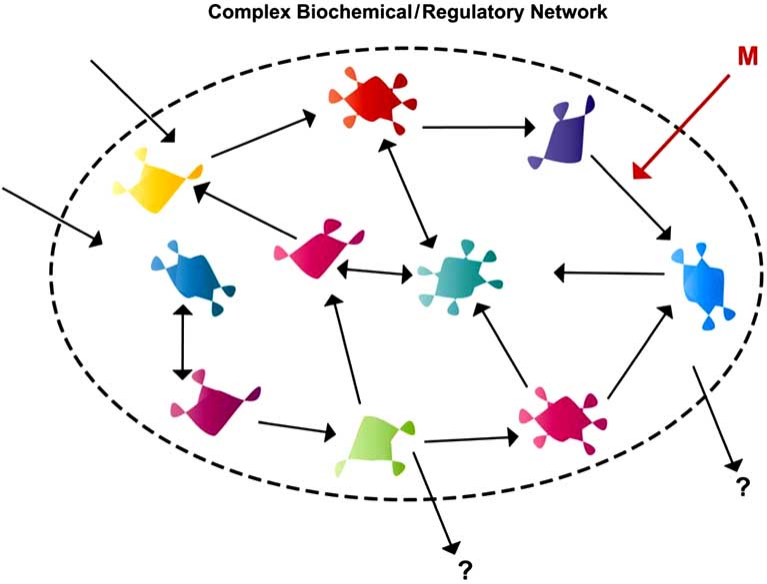
As suggested by Kacser and Burns [184], measures of enzymatic rates in purified *in vitro* systems do not necessarily reflect true *in vivo* situations. In reality, metabolic networks are complex, often with alternative routes from substrate inputs to catabolite outputs. In such cases, changing the rate of one specific step (indicated by arrow M), either *via* genetic mutation or through a targeted chemical antagonist or agonist (i.e. a drug) might lead to only modest, or unpredictable effects on total flux through the system and on the equilibrium levels of intermediate metabolites. This could explain both dominance as well as functional redundancy, whereby mutation of most genes even in homozygotes is not lethal, even though the genes are “required” in a mechanistic sense. Similar situations could be the case for developmental regulatory networks, where the flow of information rather than chemical metabolites is concerned.

The recessive nature of most mutations was appreciated early in the 20^th^ century, and theoretical geneticists developed several alternative models to explain this [181, 182]. Wright suggested that two doses of genes provided near-saturating levels of activity for the ultimate physiological trait, such that loss of one dose had a non-linear and only weak effect on the trait (Fig. **2**) [183]. Fisher suggested instead that mutant alleles evolved in concert with genetic modifiers elsewhere in the genome. Wright’s argument received strong theoretical support from the metabolic control rate theory of Kacser and Burns [184, 185]. These theoreticians sought to understand why rates of production of pathway end-products in engineered microorganisms were difficult to influence by changing amounts of individual enzymes in the reaction pathways, even when those enzymes were known to be ‘rate-limiting’ based on biochemical kinetic studies. They showed mathematically that control of flux rates through complex metabolic pathways *in vivo* was more likely to be distributed than focused on one gene product (Fig. **3**) [184]. The result would be that modest (even two-fold) changes in dosage of a gene product would be unlikely to affect overall flux through a metabolic pathway, although this point has been argued [186]. Assuming that some kind of biochemically quantitative pathways underlie all physiological traits, even those of embryonic development or neuropsychiatric function, metabolic control theory suggests at least in principle that gene dose response could readily be nonlinear, and that loss of even a full copy of gene function might have only partial effects in non-enzymatic systems [187].

Genetic studies in model organisms tend to support these arguments [168]. Although spontaneous or mutagen induced mouse mutants can span the full range of mutation types, engineered knockouts almost always involve deletions of large amounts of protein-coding potential, and are likely to function as lof alleles in most cases. Anecdotal evidence from mouse geneticists suggests that most such knockouts have no detectable phenotype in heterozygous state. A caveat to these observations of course is that mice cannot tell us what is wrong with them, thus some heterozygous knockout mice could have physiological phenotypes undetected by experimentalists. Similar results are seen in fly mutagenesis experiments, in which mutagenized flies are made heterozygous against a strain carrying a defined chromosomal deletion, with breakpoints defined either cytologically or now by DNA sequencing. In such experiments, many independent alleles can be obtained for most genes covered by the deletion, and their phenotypes compared against the deletion or when homozygosed individually or combined as compound heterozygotes against each other. The general result of such studies is that most alleles with a detectable phenotype in most genes act recessively, with no obvious heterozygous effect.

In a unique analysis to test the Wright hypothesis directly, the mutational spectrum was reviewed for the microorganism *Chlamydomonas*, which normally grows in a haploid state. When semi-synthetic diploids are generated, mutations which originally arose in haploids prove nonetheless to be almost exclusively recessive [188]. This result strongly argues against recessivity arising from coordinate evolution of oligogenic modifiers in the diploid state, and supports Wright’s argument that recessivity of mutations is a consequence of the way in which metabolic pathways have evolved. Efforts to extend the Kacser and Burns argument to other, nonmetabolic developmental pathways have shown that this is in principle feasible [181]. Nonetheless, it remains conceivable that certain types of developmental programs might not be so buffered in higher eukaryotes. One example might be the human brain. Copy number studies have documented a significantly higher incidence of CNVs, mostly deletions, in autistic than in control patients, presumably mostly in the heterozygous state. If so, then loss of function mutations might cause frequent dominant phenotypes such as schizophrenia or autism which both appear to have a high rate of sporadic etiology consistent with high mutation rates and recent evolutionary events.

Given that mutations in most genes probably act recessively, the rate of monogenic disorders ascertained clinically is substantially lower than the rate of appearance of new alleles. It depends on a combination of new mutation rates and population demographics. In outbred large populations, individuals will rarely be born carrying two non-complementing mutations in *trans* in the same gene (either as homozygotes or compound heterozygotes). In contrast, in population isolates experiencing significant inbreeding due to either geographic or cultural factors, rates of recessive disorders may be substantially higher than otherwise expected. In addition, due to founder effects different genetic disorders occur at increased frequency in different populations. This is well-documented by medical geneticists, in diverse groups such as Finns, French Canadians, Ashkenazi Jews and Alberta Hutterites [167, 189-192]. In one town in Israel, geneticists identified 19 different recessive monogenic disorders arising in a population of less than 10,000 [193].

## DEFINING THE REST OF THE PHENOME

If only a small fraction of protein-coding genes have a molecularly characterized monogenic phenotype in humans as of now, what does this mean for the complete phenome? If the complete phenome comprises all possible phenotypes of all genes in the genome, then the phenome is effectively infinite. However, if most loss-of-function alleles in a given gene cause similar phenotypes, then the lof phenome is much smaller, probably finite, and only slightly larger than the number of genes. Further, if gof alleles are much rarer than lof alleles, then most of the gof phenome will never be observed given the total human population size. In contrast, as described previously, the lof phenome probably exists across the world near completion now.

All these points together strongly suggest the following scenario: lof alleles of all genes in the genome arise regularly around the world; most of these represent recessive alleles; clinical ascertainment of homozygotes for these alleles will be individually rare except in founder or other population types with higher rates of consanguinity than expected for complete panmixis; compound heterozygotes will arise at low rates essentially everywhere, with little or no increased rate in founder populations. The actual rate of appearance of recessive cases that might be clinically ascertained is very difficult to estimate, since it depends on current allele frequencies for deleterious alleles, which in turn depend partly on details of local population demographic history which for most populations are poorly known. However, even in presumptive outbred or non-founder populations there can be increased frequency of recessive deleterious alleles through unappreciated low levels of population mobility – the reduced number of observed versus potential haplotypes found in non-African populations provides an extreme example of this.

The inescapable conclusion is that recessive cases for lof alleles of most genes do occur regularly. Where then are these missing phenotypes? Three possibilities remain: either many genes mutate to a neutral lof phenotype, or else they mutate to an embryonic lethal phenotype, or else they mutate to phenotypes not yet clinically defined or molecularly characterized as genetic conditions. Half of all spontaneous miscarriages show cytogenetically detectable chromosomal abnormalities [194]. With the advent of high density hybridization arrays, smaller rearrangements especially deletions can readily be detected, well below the resolution of light microscopy [195, 196]. It seems likely that an additional fraction of miscarriages, even earlier in fetal development, entail mutation of critical embryonic genes. For example, viable human mutation phenotypes are known for six of the 39 genes encoded by the four HOX gene clusters [197]. These are among the extreme rostrally and caudally expressed genes HOXA1 [198], A2 [199], A11 [200], A13 [201, 202], D10 [203, 204] and D13 [205]. Mutations in the majority of more centrally expressed HOX genes may mutate to recessive embryonic lethality early in development, as probably do other early expressed pattern-forming genes. OMIM currently includes several thousand phenotype entries with suggestion of a genetic etiology, many of which probably do represent recessive cases arising in families too small to map a genetic locus.

The advent of dense SNP marker panels has greatly facilitated the mapping of recessive genetic loci, especially in population isolates with not only increased recessivity but also homozygosity. Formal linkage analysis is relatively inefficient for recessive disorders unless there are an unusually large number of affected individuals in a given pedigree or related population (*i.e*. founder effects). The availability of new technologies is about to change this paradigm. In the last two years multiple individual human genomes have been sequenced to high coverage using next-generation technologies [206-213]. To reduce the cost of whole genome analysis, hybridization capture methods have been developed by which selected subsets of the genome, such as protein-coding exons, can be sequenced [214-217]. Individual genomes have millions of differences from the human consensus sequence as assembled by the Human Genome Project (current version hg19, also called GRCh37). Of the 3-4 million such differences in individual genomes, over 90% can be found in dbSNP v130, which includes a large number of variants discovered through the 1000 Genomes Project [218-220]. The large number of remaining variants is still too many to define pathogenic causality in general cases, although several recent reports demonstrate the feasibilility of array-capture exome resequencing to define likely pathogenic variants in affected individuals or families with rare clinical phenotypes under ideal conditions [221-229]. Operationally, assigning genotype/phenotype causality unambiguously requires either multiple patients mutated in the same gene with a rare monogenic phenotype, or alternatively strong validation of a candidate gene through animal model studies. Nonetheless, whole genome or exome sequencing is poised to revolutionize medical genetics, allowing the identification of causal genes for many more rare phenotypes, including some (such as prenatal lethality, or dominant disease with reduced viability or fertility) for which family studies are intrinsically impossible.

This review has focused primarily on protein coding genes. There are some examples of genes producing non-protein coding RNAs for which high penetrance mutational phenotypes occur (the H19 gene in mice [230], and the XIST gene in humans) [231-233]. In general such assignments are challenging since we have little ability to define functional changes in non-coding RNAs except when they are extensively deleted. It remains to be seen whether naturally occurring variation in other such RNAs, such as microRNAs, can lead to monogenic phenotypes. One exception is in the mitochondrial genome. Well documented mutations in mitochondrial genes, including tRNA and rRNA coding genes, lead to clinical disorders of the mitochondrion, which although complex physiologically are generally definable by experienced clinicians [234, 235].

## PRACTICAL APPLICATIONS OF PHENOME SATURATION

Beyond the purely scientific value of determining high penetrance mutational phenotypes for as many genes as possible, there is also practical value in this program. The human genetics community has focused much of its effort for the past decade on defining risk variants for complex, often adult-onset degenerative diseases of great social and commercial importance. This work has mostly employed the analytical paradigm of high throughput SNP genotyping and case/control comparison using large patient cohorts (the GWAS approach). GWAS studies have succeeded in identifying many such risk factors, but in general the variants found individually add only small incremental risk of disease, moreover only a modest fraction of total genetic risk in populations is explainable summing over all such common alleles [236]. The emerging consensus is that finding the “missing heritability” will require high throughput genomic resequencing in larger and larger cohorts. If one of the goals is to identify novel therapeutic targets for pharmaceutical intervention, this will be a long-term and monumentally expensive proposition.

In contrast, there are many good examples of high penetrance monogenic traits of direct relevance to common disease. Elsewhere I have discussed in depth the use of “monogenic disorders” as a tool to define novel therapeutic targets [237]. These can be found either by traditional family-based linkage studies [238-240], or for quantitative traits such as body mass, plasma glucose or cholesterol, by sequencing population outliers looking for excess variation in specific genes [241-245]. Essentially, some component of the missing heritability for medically important traits lies in a large aggregate number of individually rare alleles. Such genes provide “low hanging fruit” for drug development, something desperately needed by the biotechnology and pharmaceutical industries, which are universally acknowledged to be suffering a dearth of good new products. For example, the human genome encodes roughly 370 G-protein coupled receptors, not including olfactory receptors. Many of these are targets of drugs already on the market, as GPCRs have several major advantages for new drug development. As with the human genome in general, only about 10% of GPCRs have documented human monogenic phenotypes. Many of these human phenotypes are directly relevant to important disease models. Thus the GPCR phenome represents a fertile area for genetic analysis, requiring only the resequencing of this set of genes in clinically defined cases of medical interest.

Setting aside the current hype surrounding personalized genomics, there are several ways in which phenome saturation will have immediate diagnostic benefit. In pediatric hospital settings, a substantial fraction of admissions involve genetic disorders [246, 247]. Some of these, such as congenital heart malformations and neural tube defects, are seemingly genetically complex. But others involve genetically simple disorders. For example, pediatric hospitals often have dedicated cystic fibrosis units, as CF is one of the most commonly diagnosed pediatric genetic disorders with a known single gene basis. Given that there are already nearly 2500 molecular characterized genetic disorders, many of which are regularly ascertained in any large pediatric care setting, genetic analysis is critical in pediatrics even now. The definitive diagnosis of such conditions, either through molecular analyses or simply by phenotypic description, has high value to patients and families, and potentially to the health care system as well. Further definition of all remaining pediatric monogenic conditions will prove similarly useful, if only to eliminate non-genetic causes of disease in individual patients, ending otherwise time-consuming and expensive diagnostic odysseys.

## CONCLUSION

I have attempted to document that most of the human phenome remains to be ascertained and molecularly characterized. This is primarily due to challenges in clinical ascertainment and gene mapping, not to any theoretical issues with intrinsic deleterious mutation rates or the occurrence of previously uncharacterized phenotypes. Mutation rates in the human population are sufficiently high that pathogenic variants must exist in all functional genes at the present time. Most of these are likely to cause recessive phenotypes, thus ascertainment depends on identifying homozygous or compound heterozygous mutation carriers from among all medically identified patients. Historically, causal gene identification has depended on the identification of multiplex families, followed by mapping with panels of anonymous DNA polymorphisms, position cloning and resequencing. With the advent of next-generation sequencing technologies, it is now feasible to sequence whole genomes or exomes of patients without the need for families or linkage mapping. However, the large amount of individual genome variation raises the challenge of identifying causal variants in these patients. This will require collaboration among medical geneticists dispersed around the world, possibly with development of large genotype/phenotype databases, together with the use of model organisms to validate gene function and/or mutation pathogenicity.

With these ideas in mind, this is my own estimate of the lof human phenome. Time will tell how accurate these predictions are.

Known genes with phenotypes (2200-2400)

Embronic lethals (5000)

Intellectual disability or other high penetrance neuropsychiatric condition (1000)

No clinical phenotype (3000)

Phenotypes to be defined or characterized (8-10000)

## WEB SITES

Main NCBI entry site http://www.ncbi.nlm.nih.gov/

UCSC genome browser http://genome.ucsc.edu/

HGMD curated mutation database http://www.hgmd.cf.ac.uk/ac/index.php

CCDS gene annotation database http://www.ncbi.nlm.nih.gov/projects/CCDS/CcdsBrowse.cgi

Mammalian Gene Collection database http://mgc.nci.nih.gov/

Mitochondrial proteome database http://www.mitop.de:8080/mitop2/

Canadian rare genetic diseases http://raredisorders.ca/

Comparative genome annotations http://ecrbrowser.dcode.org/

GeneTests molecular diagnostic database http://www.ncbi.nlm.nih.gov/sites/GeneTests/?db=GeneTests

BioGPS gene expression database http://biogps.gnf.org/#goto=welcome

Budding yeast mutation database http://www-sequence.stanford.edu/group/yeast_deletion_ project/deletions3.html

Fission yeast mutation database http://www.sanger.ac.uk/Projects/S_pombe/

Statistics Canada http://www.statcan.gc.ca
